# In vitro maturation of oocytes from excised ovarian tissue in a patient with autoimmune ovarian insufficiency possibly associated with Epstein-Barr virus infection

**DOI:** 10.1186/s12958-018-0350-1

**Published:** 2018-04-05

**Authors:** Irma Virant-Klun, Andrej Vogler

**Affiliations:** Reproductive Unit, Department of Obstetrics and Gynecology, University Medical Centre Ljubljana, Zaloska cesta 002, 1000 Ljubljana, SI Slovenia

**Keywords:** Case report, Epstein-Barr virus, In vitro maturation, Laparoscopic biopsy, Oocyte, Ovarian failure, Vitrification

## Abstract

**Background:**

Some reports show that it is possible to isolate immature oocytes from human ovarian tissue retrieved by a cortex biopsy or ovariectomy of non-stimulated ovaries and mature them in vitro. The mature oocytes can be vitrified and stored for in vitro fertilization, which, along with ovarian tissue cryopreservation, is mostly practiced in young cancer patients to preserve their fertility. There is much less data on this new approach in women with a natural ovarian insufficiency, which can be caused by different factors, including viral infection. In this case report this advanced methodology was used in a young patient suffering from ovarian insufficiency which was possibly associated with Epstein-Barr virus and infectious mononucleosis (glandular fever).

**Methods:**

This case report included a 27-year-old patient who attended our infertility clinic because of ovarian failure as a part of autoimmune polyendocrinopathy that occurred after Epstein-Barr virus infection, which has rarely been reported until now. Although antral follicles were observed in her ovaries by ultrasound monitoring, she was amenorrhoeic with menopausal concentrations of follicle-stimulating hormone (FSH) and without mature follicles. Therefore, a small biopsy of ovarian cortex tissue was performed using laparoscopy to retrieve immature oocytes. The retrieved oocytes were matured in vitro, cryopreserved, and stored for in vitro fertilization and potential pregnancy.

**Results:**

Four immature, germinal vesicle (GV) oocytes were found and removed from tissue, denuded mechanically by a pipette, and matured in vitro in a maturation medium with added FSH and hCG as well as in co-culture with cumulus cells, which were retrieved by their denudation. Three oocytes matured in vitro to the metaphase II (MII) stage and were vitrified for in vitro fertilization along with ovarian tissue cryopreservation.

**Conclusion:**

Our results show that Epstein-Barr infection is possibly associated with autoimmune ovarian failure. The devastating impact on fertility in such disorder can be successfully avoided by in vitro maturation of oocytes from excised ovarian tissue.

## Background

Recent reports have demonstrated that it is possible to isolate antral follicles and immature oocytes from human ovarian tissue retrieved by a cortex biopsy or ovariectomy of non-stimulated ovaries and mature them in vitro [1–15]. The retrieved oocytes, which mature in vitro, can be vitrified and stored for in vitro fertilization. This is mostly practiced in young cancer patients, including those with borderline ovarian cancer [4], progressed ovarian cancer [12], and endometrial carcinoma [11], who cryostore their ovarian tissue before chemo- and radiotherapy to preserve their fertility. This cancer treatment is quite advanced and enhances the long-term survival of patients.

In vitro maturation and vitrification of oocytes retrieved from non-stimulated ovaries is usually performed along with ovarian tissue cryopreservation, which is performed to restore fertility by autotransplantation of tissue after cancer therapy [16]. It is less complicated and safer to preserve fertility in this way than autotransplantation of ovarian tissue, which cannot completely exclude the reintroduction of malignant cells into the body; future transplantation of ovarian tissue is not recommended for several cancer types, such as leukemia, due to high risks of reintroducing malignant cells [17–19]. There are also some reports on the in vitro maturation and cryopreservation of oocytes retrieved from intra-operative aspiration during cancer surgery (e.g., second enucleation of ovarian tumors) [20].

It has been shown that a significant number of immature oocytes can be collected from excised ovarian tissue regardless of the menstrual cycle phases and age of the patient, even for prepubertal girls [8]. Recent findings show that it is possible to isolate immature oocytes even from surplus ovarian medulla tissue and to mature them in vitro [21, 22]. Moreover, first pregnancies and the birth of a healthy baby achieved in this way have been reported recently, which make this new technology beneficial and promising for oncological patients and infertile women [12–14]. This new methodology can also be supported by advanced preimplantation and prenatal genetic testing.

There is not much data on this new approach in women who are infertile because of a natural ovarian insufficiency except for mosaic Turner syndrome [5] and an insufficiency following therapeutic embolization of the left uterine and right ovarian artery because of an arteriovenous malformation [13]. This case report included a 27-year-old patient who attended our infertility clinic because of premature ovarian failure (POF) which was possibly associated with Epstein-Barr virus and infectious mononucleosis (glandular fever). Epstein-Barr virus is rarely related to female infertility in the literature [23]; it may be related to altered immunophenotypic parameters and peripheral immunostimulation in women with a history of infertility [24].

## Methods

The 27-year-old patient was referred to our institution by her endocrinologist due to typical signs and symptoms of POF as a part of an autoimmune polyendocrinopathy that occurred after Epstein-Barr virus infection 3 years ago. At that time, she did not desire to become pregnant, but planned to conceive in the next couple of years. In addition to POF, she had Addison’s disease and thrombocytopenia, but normal thyroid function. For the last 2 years, she has not received hormonal replacement therapy (HRT); she was amenorrhoeic with menopausal concentrations of FSH (82.5 IU/L and 99.7 IU/L, respectively). Vaginal ultrasound examination 3 months after HRT (combination of estradiol valerate 2 mg and norgestrel 0.5 mg) withdrawal revealed a normal ovarian appearance with more than 10 antral follicles in each ovary without a dominant follicle. Genetic testing showed normal female karyotype and no fragile X-associated ovarian insufficiency (FXOI).

To retrieve ovarian tissue with possible immature oocytes, mature them in vitro, and vitrify them to perform a later in vitro fertilization along with ovarian tissue cryopreservation, laparoscopy was performed. During laparoscopy, the ovaries were normal in size with small follicles throughout the surface. With cold endoscopic scissors, a 10 × 5 mm section of the ovarian cortex was excided from each ovary opposite to the ovarian hilum. After washing the ovaries, hemostasis was achieved by placing a suture on the defect without using any thermal energy. The ovarian cortex tissue was stored in a sterile physiological saline (B. Braun, Melsungen AG, Germany) at room temperature and immediately brought into the laboratory to find possible immature oocytes, mature them in vitro, and vitrify them to perform a later in vitro fertilization along with ovarian tissue cryopreservation. At the same time a small specimen of each ovarian tissue was taken for histopathological examination. Pathology report showed active parenchyma of the right ovary with some primordial follicles and ovarian parenchyma of the left ovary with numerous primordial and some primary follicles.

This was approved by the Slovenian National Medical Ethical Committee (approval 0120–222/2016–2, KME 115/04/16), and the patient provided informed consent for voluntary participation.

### Oocyte retrieval

A section of ovarian tissue was transferred to the IVF Laboratory, placed into Universal IVF Medium (Origio, Denmark), minced by a surgical blade (Tro-Microcision, Troge, Germany) and searched for follicles and oocytes. All oocytes were removed from tissue and denuded mechanically by a denudation pipette with a diameter of 134–145 μm and length 75–85 mm (ref. 14,301, Vitrolife, Sweden). Surrounding follicular cells with predominating granulosa cells were removed easily and stored for co-culture during the in vitro maturation procedure to provide an ovarian niche to some extent. The remaining ovarian cortex tissue was cut into smaller pieces and cryopreserved using a propanediol as a cryoprotectant and finally stored in liquid nitrogen (− 196 °C) to perform in vitro activation of follicles and autotransplantation of ovarian tissue beneath the serosa of both fallopian tubes in the future [25].

### Oocyte in vitro maturation

For the maturation of oocytes, the certified MediCult IVM System (Origio, Målov, Denmark) was used, which consisted of two solutions: 1.) LAG medium (conditioning) and 2.) in vitro maturation (IVM) medium, which consisted of 100 μl of recFSH solution (75 mIU/ml; Puregon, Merck Sharp & Dohme Gmbh, Haar, Germany), 10 μl of hCG solution (100 mIU/ml; Pregnyl, Organon, Oss, Netherland) and 10 ml of basic IVM medium. Each oocyte was separately matured in a 60 μm drop of maturation medium covered by mineral oil in a UGPS-500 dish (Universal GPS® dish, LifeGlobal Group, Guilford, CT 06437, USA) at 37 °C in an atmosphere of air with 6% CO_2_ and 95% humidity. First, they were incubated for 2–3 h in the LAG medium and then for up to 48 h in the maturation medium with added FSH and hCG. Oocytes were co-cultured with granulosa cells retrieved by the previous denudation of oocytes to provide an ovarian niche to some extent; in usual practice, denuded human oocytes are matured in vitro without a granulosa cell co-culture.

## Results

**Fig. 1:**
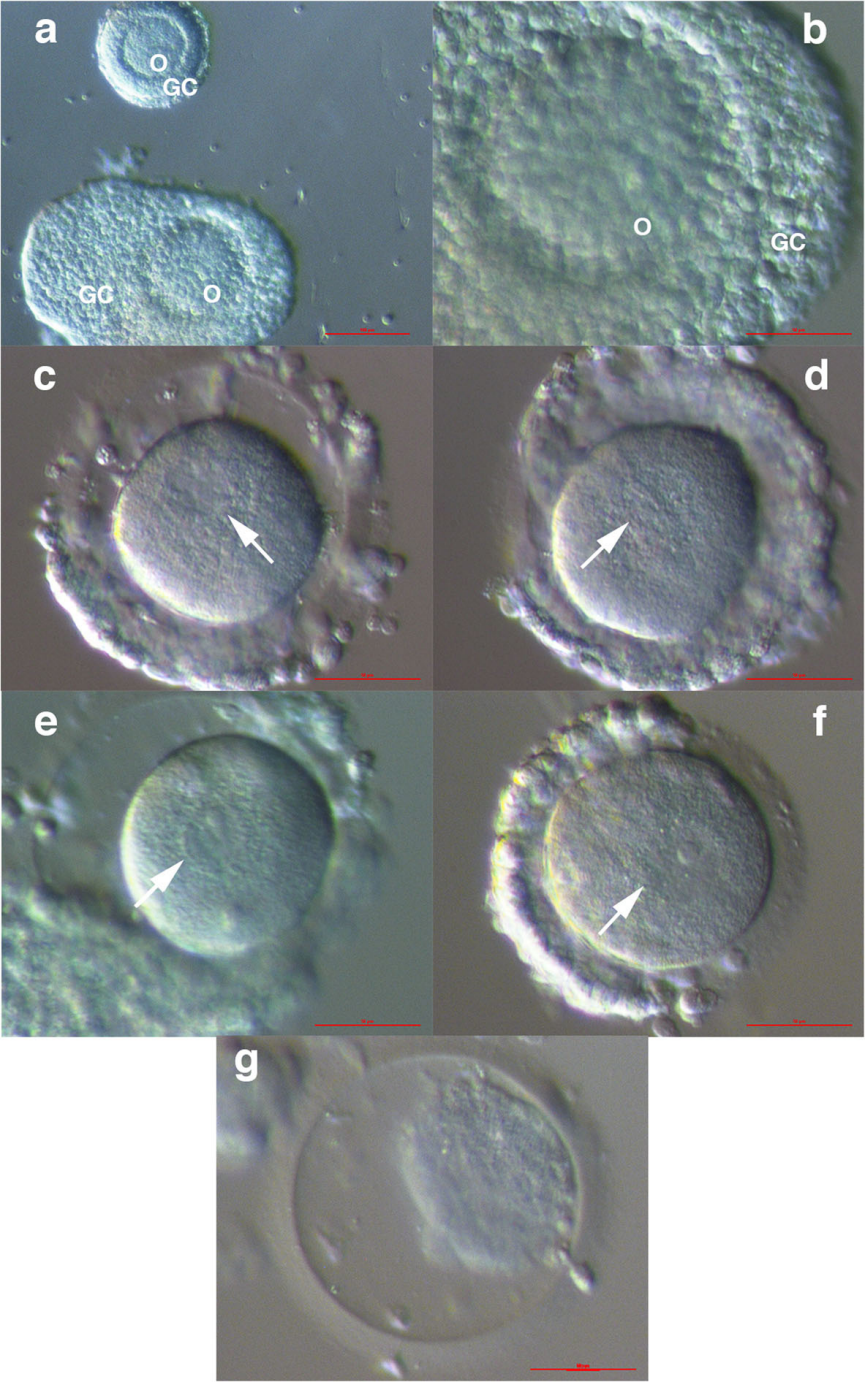
Immature oocytes expressing a germinal vesicle (arrows) and an immature follicle from the excised ovarian cortex tissue of a patient with ovarian insufficiency. Along with the immature follicle, (**a**) one oocyte appeared to have a follicle-like structure (**a, b**) and was denuded (**c**) to remove granulosa cells. Three oocytes were surrounded by a relatively thin layer of granulosa cells (**d**-**f**). One oocyte was degenerated (**g**). (Inverted microscope, magnification 40× for **a** and 100× for **b**-**g**). *Legend*: O-immature oocyte and GC-granulosa cells. *Red Bar*: 100 μm for **a** and 50 μm for **b**-**g**

Five immature germinal vesicle (GV) oocytes were found in the tissue: one oocyte was embedded in the follicle-like structure (Fig. 1a-c), similarly to a publication by Huang [5]. Three oocytes appeared with a thinner layer of follicular cells around them (Fig. 1d-f), and one oocyte was degenerated (Fig. 1g). In addition to oocytes, one immature follicle was found and isolated from the tissue (Fig. 1a).

**Fig. 2:**
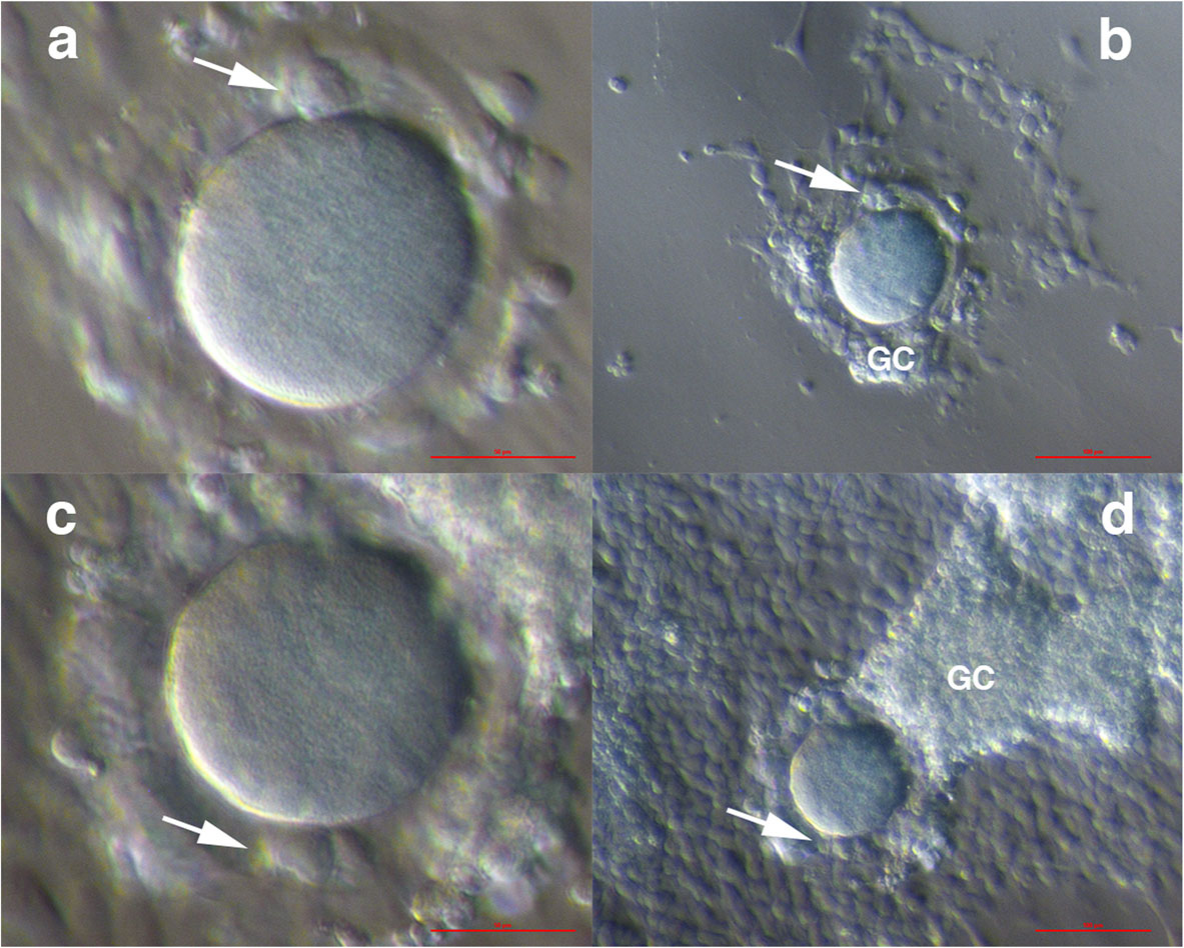
In vitro maturation of oocytes in a maturation medium with added FSH and hCG and in a co-culture with follicular (granulosa) cells retrieved by mechanical denudation of the same oocytes before in vitro maturation. Oocytes were covered by granulosa cells, forming a primitive follicle-like structure. Two oocytes matured in vitro to the metaphase II (MII) stage and extruded a polar body (arrow) (**a**-**d**). (Inverted microscope, magnification 100× for **a**, **c** and 40× for **b**, **d**). *Legend*: GC-granulosa cells. *Red Bar*: 50 μm for **a**, **c** and 100 μm for **b**, **d**

**Fig. 3:**
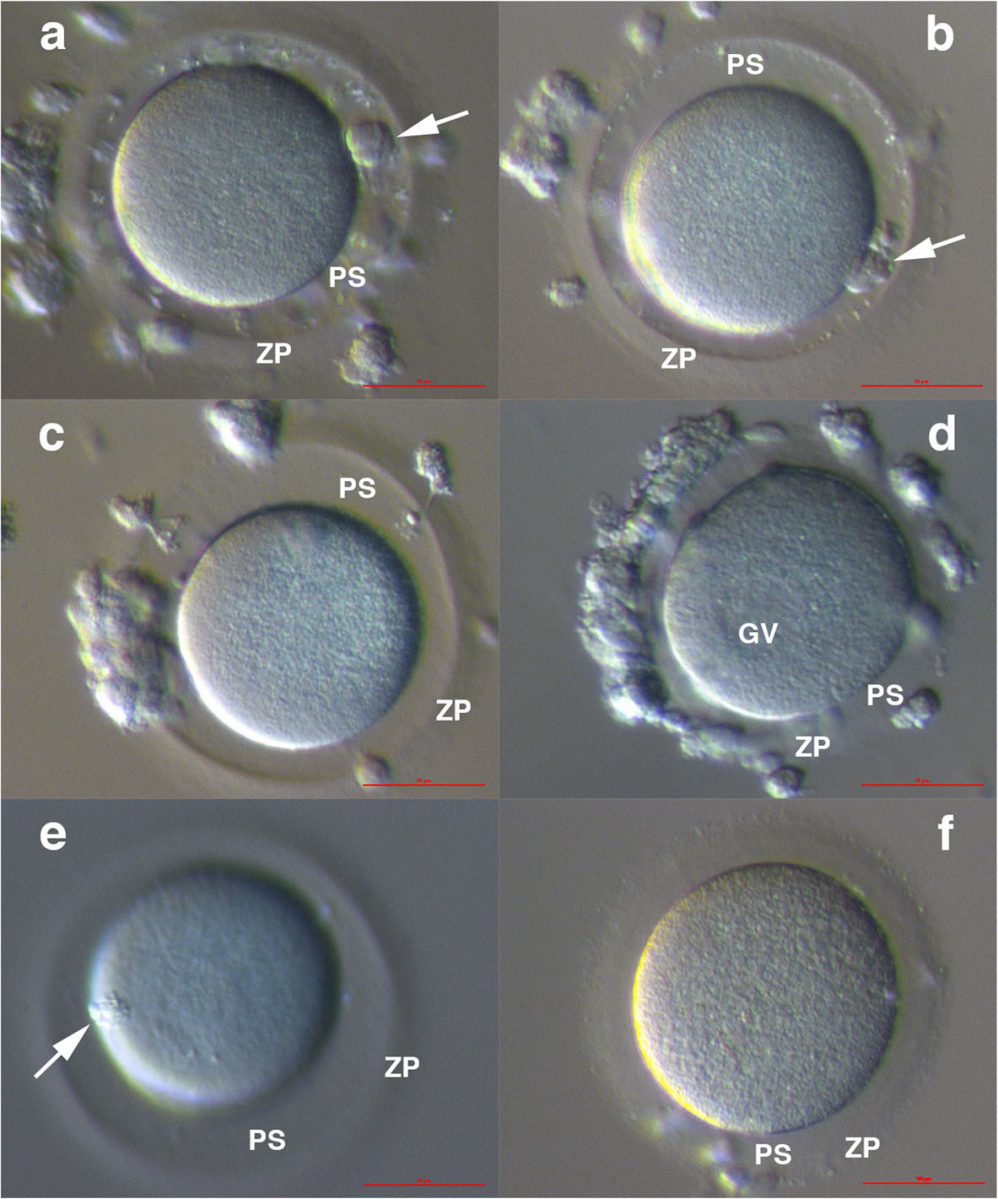
Oocytes after in vitro maturation were removed from co-cultured granulosa cells and denuded mechanically to remove the granulosa cells. Two oocytes matured to the MII stage after 38–42 h of maturation and extruded a polar body of normal morphology (arrow) (**a**, **b**), while two other oocytes did not mature in vitro over this time frame (**c**, **d**); one oocyte was at the metaphase I (MI) stage (**c**), and another oocyte continued to express a germinal vesicle (GV) (**d**). After another 6-h maturation in vitro, the MI stage oocyte matured to the MII stage and extruded a polar body (arrow) (**e**). The in vitro matured oocytes were of normal dimensions and morphology except for a slightly higher perivitelline space (PS) and thinner zona pellucida (ZP) in comparison to oocytes from the conventional in vitro fertilization procedure after the hormonal stimulation of ovaries (**f)**. (Inverted microscope, magnification 100×). *Legend*: GV-germinal vesicle, PS-perivitelline space, and ZP-zona pellucida. *Red Bar*: 50 μm

**Fig. 4:**
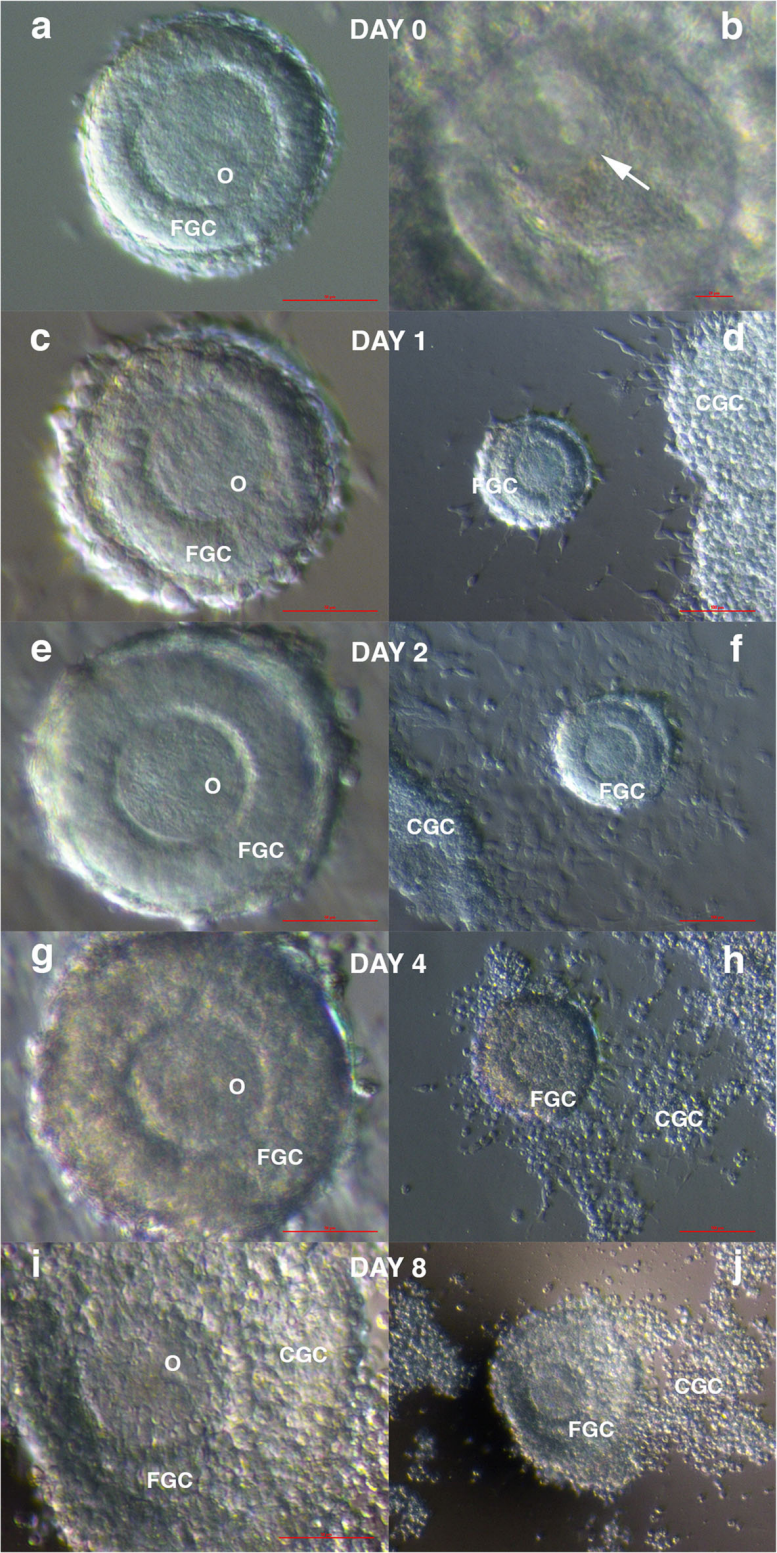
In vitro maturation of the immature follicle: the day of retrieval (**a**) of an oocyte that expressed a germinal vesicle (arrow) (**b**); on day 1, a slightly thicker layer of granulosa cells around the oocyte (**c**) in the co-culture with granulosa cells that approached the follicle (**d**); on day 2, a significantly thicker zona pellucida (**e**) in the co-culture with granulosa cells that spread over the follicle (**f**); on day 4, a thicker layer of granulosa cells around the oocyte (**g**) in the co-culture with granulosa cells, which started to incorporate into the layer of the granulosa cells around the oocyte (**h**); and on day 8, a massive layer of granulosa cells around the oocyte (**i**) in the co-culture with granulosa cells, which was massively incorporated into the layer of granulosa cells around the oocyte (**j**). (Inverted microscope, magnification 200× for **b**, 100× for **a**, **c**, **e**, **g**, **i**, and 40× for **d**, **f**, **h**, **j**). *Legend*: O-oocyte, FGC-granulosa cells around the oocyte in the follicle and CGC-co-cultured granulosa cells retrieved by denudation of oocytes in the patient. *Red Bar*: 10 μm for **b**, 50 μm for **a**, **c**, **e**, **g**, **i**, and 100 μm for **d**, **f**, **h**, **j**

Oocytes were matured in vitro. During the maturation process, cumulus/granulosa cells spread over the oocytes, forming primitive follicle-like structures (Fig. 2). Two out of four oocytes matured in vitro to the metaphase II (MII) stage and extruded the first polar body between 38 to 42 h of incubation in the maturation medium (Fig. 3a, b). These two oocytes were vitrified (Kitazato Cryotop®Oocyte Vitrification System, Japan) and stored in liquid nitrogen (− 196 °C) to be used for in vitro fertilization in the future. The remaining two oocytes did not mature in vitro (Fig. 3c, d) and were further matured in vitro for up to 48 h. One of the oocytes matured and extruded the polar body (Fig. 3e); it was also vitrified and stored in liquid nitrogen for a later in vitro fertilization. Another oocyte did not mature at all and was discarded. All of the in vitro matured oocytes that were vitrified had normal polar bodies as well as normal dimensions (diameters of ≈ 100 μm), normal shape and cytoplasm granularity in comparison with oocytes retrieved after controlled ovarian stimulation in the usual in vitro fertilization program (Fig. 3f). The immature follicle was matured in the same maturation medium, but did not mature (Fig. 4). The vitrified oocytes will be stored in liquid nitrogen for 10 years. If not used by a patient during this time period, the oocytes will be further stored or discarded after her written consent. After laparoscopy the hormonal status of patient did not change.

## Discussion

Our data show that infection with Epstein-Barr virus, also known as human herpesvirus 4, possibly triggered the polyendocrinopathy (most likely autoimmune) including POF. The devastating impact on fertility in such disorder can be successfully avoided by in vitro maturation of oocytes from excised ovarian tissue.

Ovarian insufficiency or premature ovarian failure (POF) is characterized by loss of function of the ovaries before age 40 characterized with amenorrhoea, hypergonadotropism and hypoestrogenism being one of the most serious causes of infertility [26]. It affects approximately one out of 100 women. Only 5–10% of women diagnosed with POF can achieve a spontaneous pregnancy, while most of them require medical intervention [27]. There is no generally accepted treatment to increase fertility in women with ovarian insufficiency, and the use of donor eggs with in vitro fertilization (IVF) and adoption remain the only options for achieving parenthood for women with POF. The etiology of ovarian insufficiency is still poorly understood, and most cases remain unexplained. According to our data the infection with Epstein-Barr virus with infectious mononucleosis (glandular fever) and autoimmune polyendocrinopathy is considered to be a potential trigger of ovarian failure.

The majority of the human adult population is infected with Epstein-Barr virus, and the majority of infected individuals tolerate the infection well, without any further symptoms after primary infection [28]. Normally, the virus stays in the body and remains dormant throughout life. However, this is not always the case, and a serious Epstein-Barr virus-related illness may develop later in life, especially different autoimmune diseases, such as systemic lupus erythematosus, multiple sclerosis, rheumatoid arthritis, Sjögren’s syndrome, autoimmune hepatitis, and autoimmune thyroid disorders [29]. Although this virus is not the only agent responsible for the development of autoimmune diseases, it can be considered to be the main contributory factor.

According to our knowledge, there is not much data on the association between Epstein-Barr virus infection and autoimmune ovarian insufficiency in the literature, but POF is quite frequently associated with Addison’s disease which can be in rare cases caused by the virus [30–32].

The patient included in this study had primordial and primary follicles in her ovaries, which did not mature and produce mature oocytes. By performing in vitro maturation of oocytes from excised ovarian tissue, we avoided an autoimmune disorder and cryopreserved the mature oocytes for a later in vitro fertilization along with ovarian tissue cryopreservation. This approach can be beneficial also for other patients with autoimmune POF and non-maturing follicles/oocytes in the ovaries to preserve their fertility, while it cannot help patients with POF and without follicles in the ovaries. The most limiting factor is conventional oocyte in vitro maturation procedure in the presence of reproductive hormones only, which is suboptimal and results in a relatively low number of matured oocytes. Therefore the maturating oocytes in this patient were co-cultured with cumulus cells to provide an ovarian niche as well as with substances released by cumulus cells, which are important for human oocyte maturation [33] and are usually lacking when oocytes are matured in vitro in a conventional way without a co-culture.

## Conclusion

Infection with Epstein-Barr virus with severe infectious mononucleosis (glandular fever) and autoimmune polyendocrinopathy need to be considered to be possibly associated with ovarian failure. Further histological, animal and molecular studies will be required for a final conclusion.

As in the patient included in this study, the ovaries of women with autoimmune POF need to be carefully examined for the presence of follicles because it is possible to isolate immature oocytes from excised ovarian tissue, mature them in vitro and vitrify and store them for a later in vitro fertilization along with ovarian tissue cryopreservation. The patient suffering with POF can safeguard her fertility by cryopreserving these excised oocytes. Co-culturing maturing oocytes with cumulus cells may be beneficial to provide an ovarian niche during the maturation process and improve the quality of matured oocytes.

## Abbreviations

FSH: Follicle-stimulating hormone
GV: Germinal vesicle (immature oocyte)
hCG: Human chorionic gonadotropin
IVM: In vitro maturation
MII: Metaphase II stage (mature oocyte)
POF: Premature ovarian failure
